# Commentary: The Evolution of Musicality: What Can Be Learned from Language Evolution Research?

**DOI:** 10.3389/fnins.2018.00640

**Published:** 2018-09-19

**Authors:** Rie Asano, Uwe Seifert

**Affiliations:** Systematic Musicology, Institute of Musicology, University of Cologne, Cologne, Germany

**Keywords:** computational neurocognitive modeling, music evolution, internal mechanisms, mechanistic explanation, integrated approach, evolving brain, theory-driven modeling, top-down approach

Music evolution research requires an integrated approach combining experimental research and formal-mathematical modeling, especially computer simulation of cognitive and neural processes (Anderson, 2014). In their valuable methodological contribution, Ravignani et al. (2018) reminded us of the importance of an integrated approach. We agree that musicological research is indeed in need of an *integrated methodology*. However, such a methodology is not limited to a purely inductive-probabilistic approach of the empiricist, externalist tradition examining behavioral changes. Thus, we suggest to complement Ravignani's and colleagues' data-driven bottom-up approach from the perspective of cognitive musicology and comparative biomusicology by a theory-driven top-down approach investigating the biological foundations of the human music capacity as the study of a computational (neuro)cognitive system. Especially, by introducing *computational neurocognitive modeling* as a complementary approach which explores the evolving mind/brain within a biological framework, we highlight those aspects which Ravignani and colleagues almost completely neglect such as (1) internal mechanisms, i.e., cognitive and neural processes, and representations, (2) the evolving brain, and (3) theory-driven modeling or hypothesis building.

The most appropriate biological framework to investigate language and music evolution, as already suggested by several authors (e.g., Asano and Boeckx, 2015; Fitch, 2018), is provided by Tinbergen's four questions (Tinbergen, 1963): Mechanisms/causality: How does the steady state of the mind/brain, i.e., its functional architecture and its processing mechanisms (Shallice and Cooper, 2011), look like? Ontogeny: How does a system in interaction with its environment develop from its initial state to its steady state? Phylogeny: How did that species-specific initial state evolve? Function: Why did it evolve? Importantly, Tinbergen's term “mechanism” refers to *internal causal processes or causes* underlying behaviors. This indicates that music evolution research must take internal components of the functional architecture of the neurocognitive system seriously instead of observing or experimenting with external *musics* in terms of animal signals and behaviors (e.g., Honing, 2018). These internal mechanisms are investigated at an intermediate level of research which deals with cognitive and neural processes as computations on internal representation underlying animal signals and behaviors (Figure 1).

**Figure 1:**
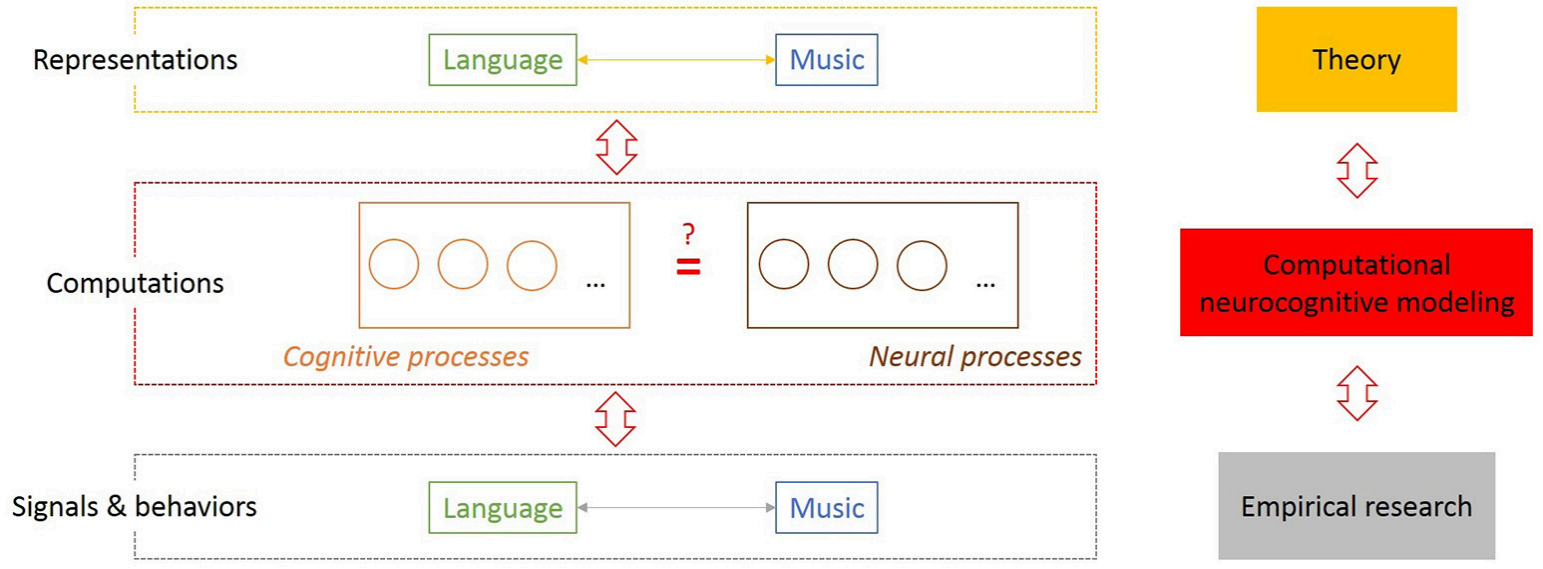
Computational neurocognitive modeling as a bridge between theory and empirical research as well as mind and brain in evolutionary research on the music capacity. This figure illustrates three levels of investigating neurocognitive systems and the mapping problem between mind and brain (Poeppel and Embick, 2005) or the explanatory gap (Levine, 1999) indicated with a question mark between cognitive and neural processes. The levels on the left and right side correspond to each other. Theories and hypotheses about (internal) representations based on intuition **(Top)** and quantified observable data on (animal) signals and behaviors **(Bottom)** are traditionally often opposed approaches in language evolution research and linguistics (Culicover and Jackendoff, 2010; Gibson and Fedorenko, 2010). Alongside the central hypothesis of cognitive science (Gallistel and King, 2009), a set of neurocognitive processes which execute computations are proposed as an intermediate level.

Although we admit that interaction and external factors need to be taken into account in research on language and music evolution (e.g., the dual-inheritance model by Richerson and Boyd, 2005; niche construction by Laland et al., 2013), we find it critical that cognitive and neural processes of the interacting computational agents and their relation to phenomenal experience, which Ravignani and colleagues avoid so deliberately, stay in focus of the approach of cognitive science and computational neuroscience to the mind/brain (Gallistel and King, 2009; Anderson, 2014). We use “computation” and “computational” as in automata theory and computability theory in the abstract sense of Turing-machine computability (Nelson, 1968; Cooper, 2004). Turing-machine computability provides a conceptual foundation with methodological implications for the research paradigms within cognitive science (cognitivism, connectionism, and dynamicism). In computer simulations of neurocognitive processes, this leads to different modeling strategies using logic, linear algebra, or differential equations.

In addition, evolutionary neuroscience is neglected although language evolution research already profited from and keeps pushing this approach (e.g., Deacon, 1997; Arbib, 2012 and many others). Its relation to biocultural coevolution research was also extensively discussed in neuroarchaeology (Stout and Hecht, 2017). Therefore, we suggest to put the evolving and developing mind/brain and *computational neurocognitive modeling* in the focus of an *integrated approach* (Poggio, 2012). This allows music evolution research to move toward a computational neuroethology or biology (Fitch, 2014; Arbib, 2016).

Dominey and colleagues' connectionist modeling approach (starting with Dominey and Inui, 2009) might serve as an example of potentially fruitful computational neurocognitive models for language and music evolution research. Their models carry out computations, i.e., transforming strings to meaning representations, on the basis of functional hypotheses. These hypotheses concern the neural implementation of specific cognitive processes required for thematic role assignment in the cortico-basal ganglia-thalamocortical (CBGT) circuits. The simulations replicated the findings of neuroimaging and neuropsychological studies. Their models provide rich comparative options by implementing hypotheses about the processing of neural circuits which are also involved in and necessary for musical rhythm processing (Kotz et al., 2009; Leow and Grahn, 2014) and are extensively studied in other animals (Jarvis, 2004; Fee and Goldberg, 2011; Mendoza and Merchant, 2014).

Several agent-based signaling game approaches such as iterated learning models as mentioned by Ravignani and colleagues and simulations of embodied agents such as Beuls and Steel (2013) made valuable contributions to language evolution research (but see Hauser et al., 2014). However, a computational Bayesian approach is not, as the article suggests, the only game in town to investigate evolutionary relevant learning mechanisms (Sun, 2008). In addition, there are serious theoretical problems with such an approach (e.g., Bowers and Davis, 2012; Glymour, 2015). We stress again that for an integrated approach computational modeling should focus on *internal mechanisms and mechanisms for (socially) interacting brains* as, for example, suggested by Arbib (2016, 2017) with a *dyadic-brain modeling approach to imitative learning* in computational comparative neuroprimatology.

Moreover, epistemologically and methodologically, scientific hypothesis formation cannot be equated with finding patterns in data, with prediction, and with law-like generalizations. Rather, it is a theory-driven hypothesis forming process with computational-representational theories as working hypotheses of the human music capacity that is required at the first place to restrict the “search space” to find mechanistic explanations for capacities underlying effects and patterns (see also McPherson, 2001; Bechtel, 2008). Figure 1 indicates the methodological levels between empirical research and theoretical reasoning with computational neurocognitive modeling at the center as necessary to bridge the gap between theory and empirical research in music evolution research and to deal with the explanatory gap (see Figure 1). The take-home message is: scientific hypothesis formation is more than statistical hypothesis formation, computational modeling is more than data fitting, explanation is more than prediction, and mechanistic explanations are more than finding covering-law explanations for regularities in data (Cummins, 2000). If we take off empiricist's blinders, then there is not only much more to learn for music evolution research from language evolution research, but also from (formal) epistemology and musicology.

## Author contributions

All authors listed have made a substantial, direct and intellectual contribution to the work, and approved it for publication.

### Conflict of interest statement

The authors declare that the research was conducted in the absence of any commercial or financial relationships that could be construed as a potential conflict of interest.
